# A retrospective epidemiological review of maxillofacial injuries in a tertiary care centre in Goa, India

**DOI:** 10.1016/j.cjtee.2022.12.004

**Published:** 2022-12-10

**Authors:** Purva Vijay Sinai Khandeparker, Trishala Bhadauria Fernandes, Vikas Dhupar, Francis Akkara, Omkar Anand Shetye, Rakshit Vijay Sinai Khandeparker

**Affiliations:** Goa Dental College and Hospital, Bambolim 403202, Goa, India

**Keywords:** Maxillofacial injuries, Epidemiology, Fractures

## Abstract

**Purpose:**

Trauma accounts for the leading cause of morbidity and mortality worldwide in the present day and may rightly be called the new pandemic. The prominent nature of the face exposes it to various traumatic injuries. A timely, prompt diagnosis along with employment of correct and quick treatment greatly improves the outcome for these patients. The aim of this retrospective study was to analyse the characteristics of maxillofacial injuries over a decade.

**Methods:**

The data were collected manually from the medical records of patients who reported to the tertiary centre from 1 January 2011 to 31 December 2019. All injured patients irrespective of age/gender with complete hospital records of clinical and radiographical diagnosis of maxillofacial injuries were included. The demographic data, etiology, site and type of injury, and seasonal variation were analyzed. Data were tabulated into 6 age groups (0 – 7 years, 8 – 18 years, 19 – 35 years, 36 – 40 years, 41 – 59 years, and > 60 years). Five etiological factors, i.e. road traffic accidents, falls, assaults, sports-related, and occupational accidents, were further evaluated based on genders. Facial injuries were classified into 6 types: panfacial fractures, mandibular fractures (subcategorized), midface fractures (subcategorized), dentoalveolar fractures, dental injuries, and soft tissue injuries. The monthly and seasonal variation of the injuries was also charted. Data were expressed as frequency and percent.

**Results:**

A total of 10,703 maxillofacial injuries were included from the tertiary centre from the period of 2011 – 2019, including 8637 males and 2066 females, with the highest occurrence in the 19 – 35 years age group. Road traffic accident was the principal etiological factor of maxillofacial injuries in both genders (80.5%), followed by falls (9.6%), assaults (8.0%), occupational accidents (1.2%), and sporting injuries (0.7%). Midface fractures accounted for 52.5% (5623 fractures), followed by mandibular fractures (38.1%).

**Conclusion:**

The current study describes a change in the incidence of maxillofacial injuries along with variation in the demographic data. The implementation of safety gears and stricter traffic laws along with public awareness may aid in the reduction of maxillofacial injuries.

## 1 Introduction

Trauma continues to be the primary cause of death occurring in the first quadragenarian of life.^1^ As of 19 March 2021, World Health Organization statistics reveal that both unintentional and violence-related injuries contribute to the mortality of 4.4 million people globally each year. These injuries claim the lives of 3.16 million people (unintentional) and 1.25 million people (violence-related) annually.^2^

The protruding nature of the facial skeleton makes it less invincible to trauma. The global variation in the distribution of maxillofacial injuries is observed in terms of topography, community, ethnicity, and habitat.^3^ The early recognition of the interrelationship between the diagnosis, severity, and complex pattern of maxillofacial injuries aids in subsequent effective management.^4^

This retrospective descriptive epidemiological study was conducted over a span of 9 years and aims to understand the occurrence of maxillofacial injuries concerning age, gender, etiology, fracture patterns, site, and seasonal changes, and to analyse the variations in maxillofacial injuries over time.^5^

## 2 Methods

The authors carried out an uni-centric observational study in the department of oral and maxillofacial surgery, Goa Dental College and Hospital, India. The institutional ethics committee approval was obtained [GDCH/IEC/VII-2022(04)-PROV]. This study was based on manual search and extraction of data from the medical records of patients who reported to the outpatient department of oral and maxillofacial surgery, Goa Dental College and Hospital, as well as the emergency department of Goa Medical College and Hospital for a time span of 9 years (1 January 2011 to 31 December 2019). The data to be included was for a decade; however, considering the global pandemic, data collection was restricted to 9 years to avoid bias arising out of the lockdown. Patients were evaluated for any maxillofacial injuries irrespective of age and gender. All injured patients irrespective of age/gender with complete hospital records of clinical and radiographical diagnosis of maxillofacial injuries constituted the inclusion criteria of the study. Patients with incomplete records were excluded.

The data were tabulated under the following categories: age, gender, etiology, the month of injury, geographic distribution, site of facial fractures, associated soft tissue, and dentoalveolar injury. The patients were assigned into 6 groups based on the age: 0 – 7 years, 8 – 18 years, 19 – 35 years, 36 – 40 years, 41 – 59 years, and > 60 years. The etiological factors were classified into the following categories: road traffic accidents (RTAs), falls, assaults, sports-related, and occupational accidents. The monthly variation of the injuries was also charted.

Radiographic evaluation of the fractures was done using 2-dimensional radiographs (paranasal sinus view, submentovertex view, posteroanterior mandible view, lateral oblique view, reverse towne's view, orthopantomogram) and CT scan with axial, sagittal and coronal sections along with 3-dimensional reconstructions.

Facial injuries were classified into 6 types: panfacial fractures, mandibular fractures, midface fractures, dentoalveolar (DA) fractures, dental injuries, and soft tissue injuries. Panfacial fractures are defined as fracture patterns that involve at least 3 of the 4 axial segments of the facial skeleton: frontal, upper midface, lower midface, and mandible. Fractures of the mandible (either isolated or comminuted) were further subdivided into the following subtypes: symphysis, parasymphysis, body, angle, ramus, coronoid, and condyle. Comminuted fracture is defined as the presence of multiple fracture lines resulting in many small pieces within the same area of the mandible. If the fracture occurred at 2 or more different sites in the mandible at the same time, such as condyle and symphysis, it was included under a separate subtype: combined/multiple.

Midface fractures comprised of the following subtypes: zygomaticomaxillary complex (ZMC) and LeFort I/II/III fractures. Fractures involving only the DA component were considered as a separate entity. Dental injuries consisted of any minor to major injury to the tooth, as per Ellis and Davey's classification. Minor superficial abrasion to deep injuries (lacerations, contusions), including avulsion of the tissue, was incorporated into soft tissue injuries.

## 3 Results

Altogether 10,703 patients met the inclusion criteria for this study. The results of the study are reported below.

### 3.1 Age distribution

**Fig. 1:**
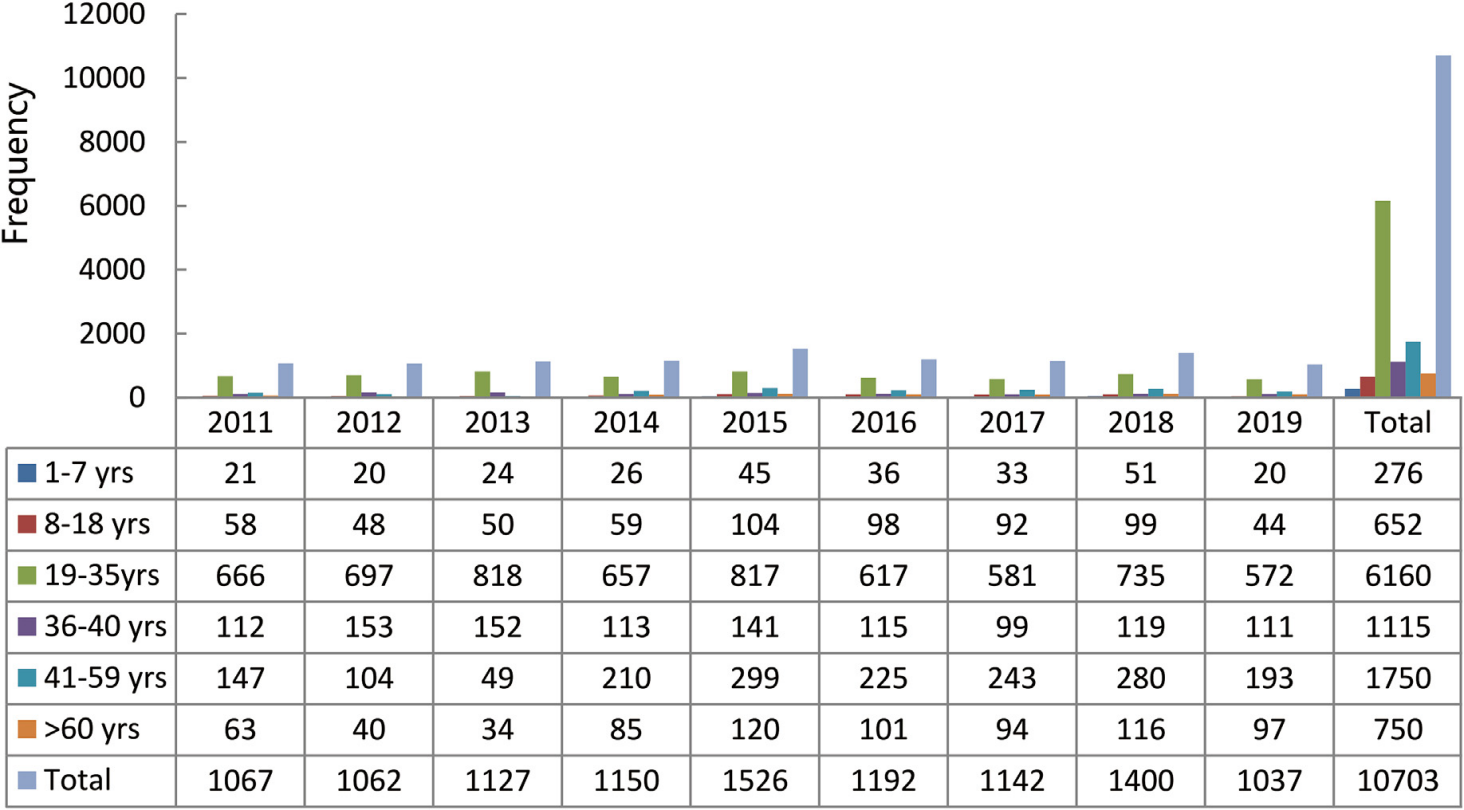
Age groups *vs.* yearly distribution. yrs: years.

The cumulative 9-year data analysis revealed that the age group of 19 – 35 years had the highest number of maxillofacial injuries, accounting for 57.6% of the entire population. It also showed an increasing trend in the age-wise distribution of injuries from the first (1 – 7 years) to the third group (19 – 35 years), followed by a decrease in the incidence in the fourth group (36 – 40 years), a slight increase in the fifth group (41 – 59 years), and a drop-down in the sixth group (> 60 years) based on the age distribution. From the year 2011, a remarkable change was seen in the frequencies of all the age groups in 5/9 recorded years, i.e. 2013, 2015, 2016, 2018, and 2019. An obvious rise was observed in all the age groups from the years 2011 – 2015, whereas a reverse was seen from 2015 to 2019. The year 2016 witnessed a marked decline in overall injuries followed by a prominent rise in the year 2018, and a noticeable drop in the following year among all the age groups, as listed in Fig. 1.

### 3.2 Gender distribution

**Fig. 2:**
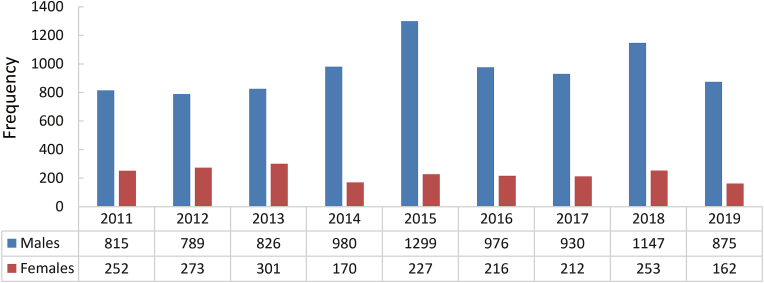
Gender-wise *vs.* yearly distribution.

Among all the patients, there were 8637 (80.7%) males and 2066 (19.3%) females, as presented in Fig. 2. An overall increase was noticed in male population over the study period, with the most prominent elevation in the years 2015 (*n* = 1299) and 2018 (*n* = 1147). A reverse phenomenon was perceived among the females. There was a clear lessening in the overall female population during the study period. However, there were 3 steady rises seen in the female population in the years from 2011 to 2013, 2014 – 2015 and 2016 – 2018 and a visible decline can be noted in 2 instances in the years 2013 – 2014 and 2018 – 2019.

### 3.3 Etiology

The collective data analysis recorded RTAs as the principal cause of maxillofacial injuries in both genders (*n* = 8615, 80.5%), with 6989 affected males (80.9%) and 1626 affected females (78.7%). Falls, either accidental or falls from height (*n* = 1027, 9.6%) were reported to be the secondary cause. This was followed by assaults (*n* = 860, 8.0%) and occupational accidents (*n* = 126, 1.2%). The least frequent cause was sports-related injuries (*n* = 75, 0.7%).

#### 3.3.1 Male population

**Fig. 3:**
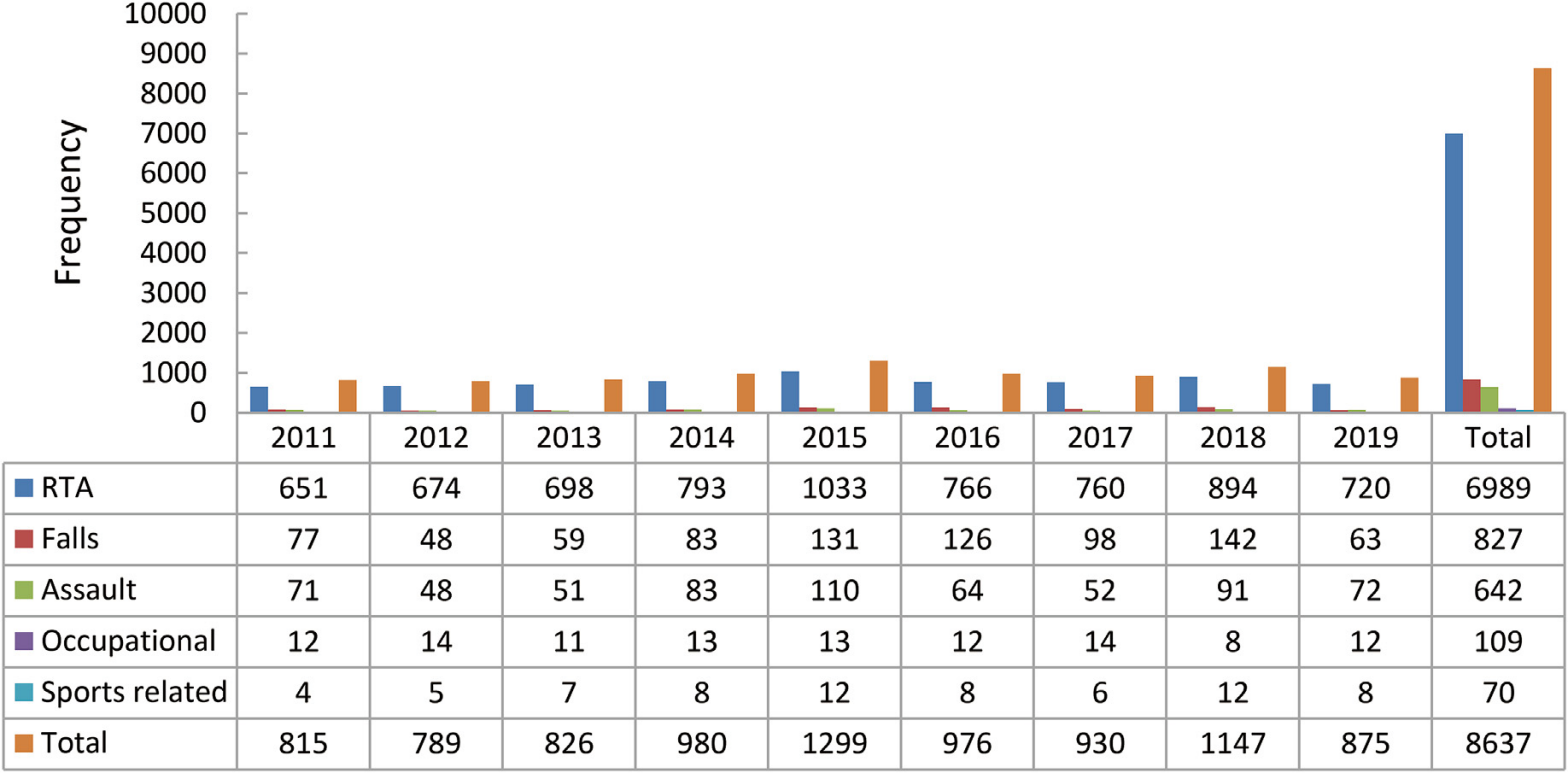
Gender-wise distribution of etiology (males). RTA: road traffic accident.

RTAs, the primary cause of trauma in male population, accounted for a marked elevation in the affected males in the years from 2011 to 2015, followed by a progressive diminution from the year 2015 – 2019. However, there was a noticeable rise in the affected males noted in the 9-year recorded data. Falls, the secondary cause, amounted to 9.6% of the total affected male population, with the maximum cases reported in the year 2018. The year 2015 witnessed the highest number of assault cases, along with RTAs and sports-related injuries. The occupational injuries reported in the years 2011 and 2019 remained the same, despite the mixed pattern of incidence in between. The detailed distribution is shown in Fig. 3.

#### 3.3.2 Female population

**Fig. 4:**
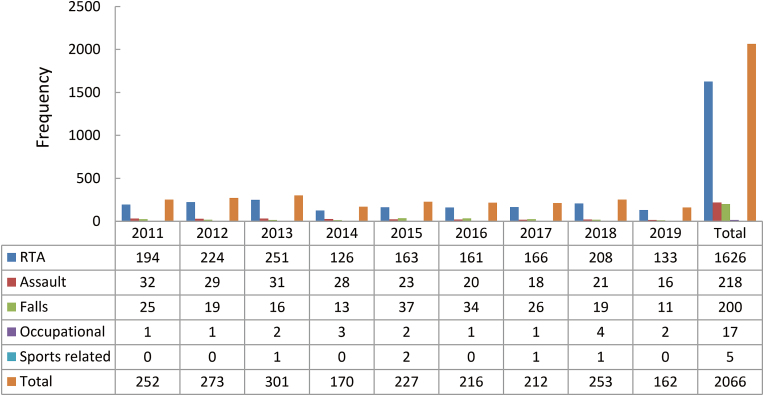
Gender-wise distribution of etiology (females). RTA: road traffic accident.

The chief cause of maxillofacial injuries in the female population was RTAs, revealing a steady ascent in the years from 2011 to 2013, 2014 – 2015, and 2016 – 2018 and a visible fall was noted in the years from 2013 to 2014 and from 2018 to 2019. The second most important cause of trauma affecting the female population was assault (*n* = 218, 10.6%). The 9-year recorded data revealed a progressive drop in the reported injuries over the years for RTAs, assaults, and falls in affected female population. The overall change in the occupational and sports-related injuries was not significant in the recorded data. The detailed distribution is mentioned in Fig. 4.

### 3.4 Fracture distribution

**Fig. 5:**
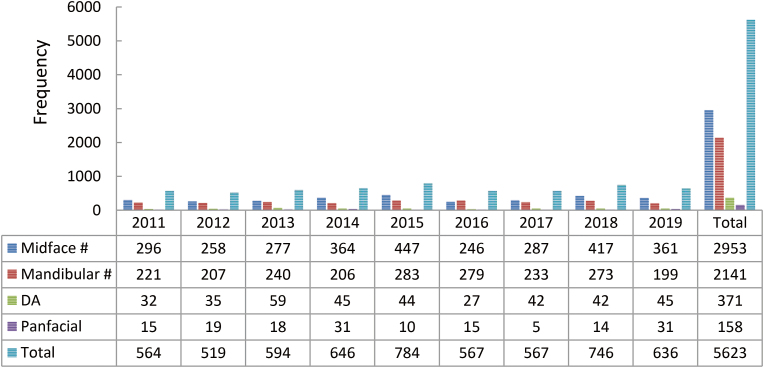
Fracture site distribution. DA: dentoalveolar.

A total of 5623 fractures were reported (Fig. 5). Of all the facial bones, the midface fractures (*n* = 2953) accounted for 52.5% of the facial injuries, followed by mandibular fractures (*n* = 2141, 38.1%), and DA fractures (*n* = 371, 6.6%). The least common fracture was panfacial fracture (*n* = 158) with a percentage of 2.8%. From the years 2011 – 2019, there were noticeable elevations in the frequencies of the midface fractures, DA fractures, and panfacial fractures with a mild reduction in the mandibular fractures frequency. The maximum incidences of mandibular and midface fractures were reported in the year 2015, DA fractures in the year 2013, and panfacial fractures in the years 2014 and 2019.

**Fig. 6:**
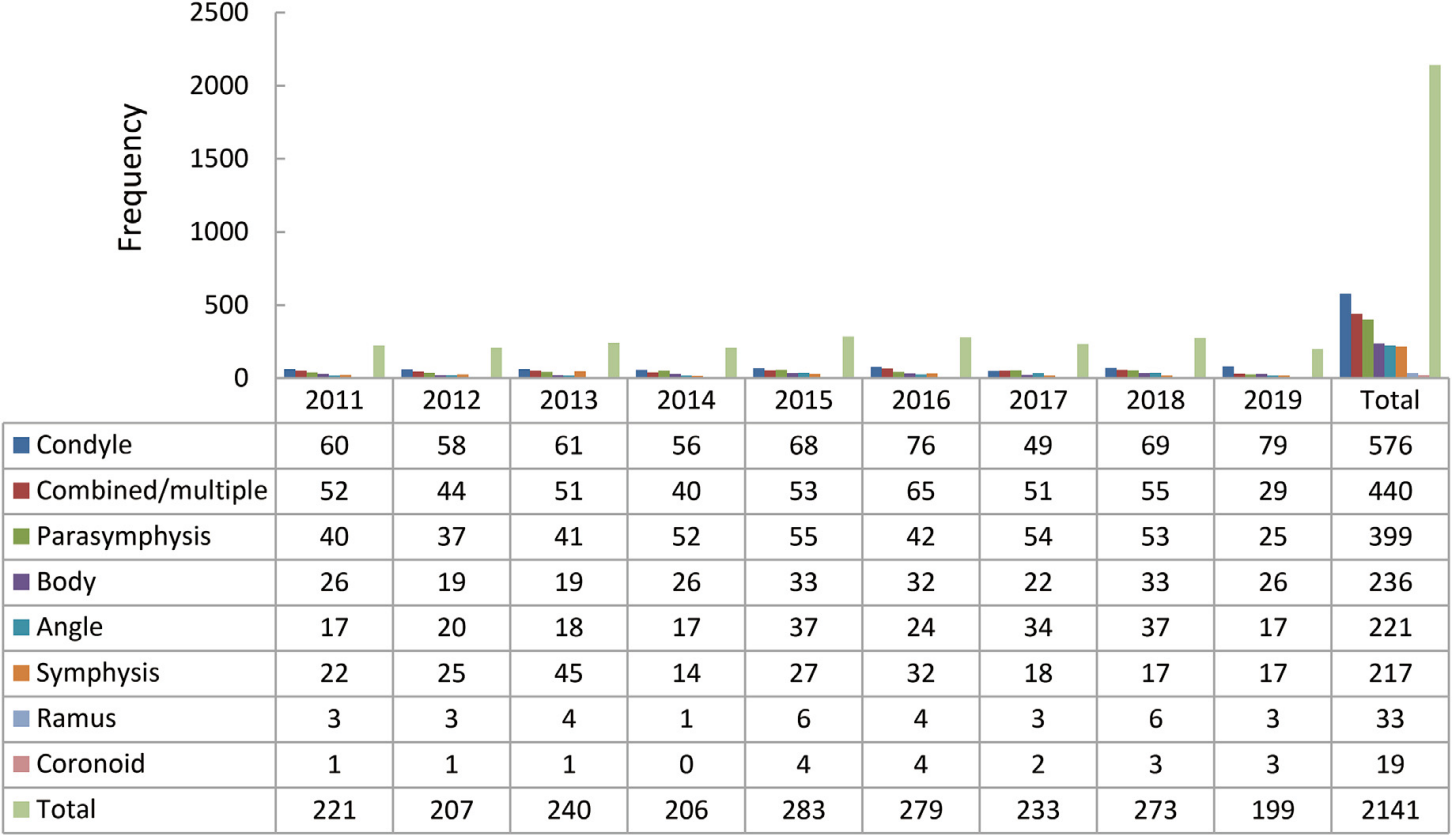
Mandibular fracture distribution.

The most common site for mandibular fractures was the condyle (*n* = 576, 26.9%) followed by combined/multiple fractures (*n* = 440, 20.6%), parasymphysis fractures (*n* = 399, 18.6%), body fractures (*n* = 236, 11.0%), angle fractures (*n* = 221, 10.3%), symphysis fractures (*n* = 217, 10.1%), ramus fractures (*n* = 33, 1.5%), and coronoid fractures (*n* = 19, 1.0%). Over the period of the study, a mild increment was seen in the overall distribution of the coronoid fractures. The highest peaks of an increase in the condylar fractures were recorded in the years 2014 – 2015, 2017 – 2018, and 2018 – 2019. There was a progressive decline noted in the distribution of combined/multiple, parasymphysis, and symphysis over the recorded period. The incidence of the body, angle, and ramus remained unchanged in the years 2011 and 2019, with mixed incidences of rise and fall in between. The yearly distribution of the mandibular fracture site is mentioned in Fig. 6.

**Fig. 7:**
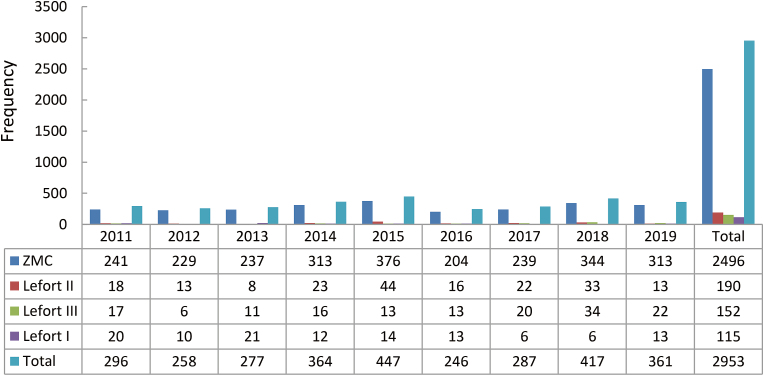
Midface fracture distribution. ZMC: zygomaticomaxillary complex.

The most common midface fractures were ZMC fractures (*n* = 2496), accounting for 84.5%. This was followed by LeFort II fractures (*n* = 190, 6.4%), LeFort III fractures (*n* = 152, 5.2%) and LeFort I fractures (*n* = 115, 3.9%). The year 2015 reported the highest occurrence of both ZMC and LeFort II fractures. The cumulative data analysis reported a considerable increase in the distribution of ZMC and LeFort III fractures; whereas a mild reduction was reported in both LeFort II and LeFort I fracture distribution. The yearly distribution of the mid-face fracture site is recorded in Fig. 7.

**Fig. 8:**
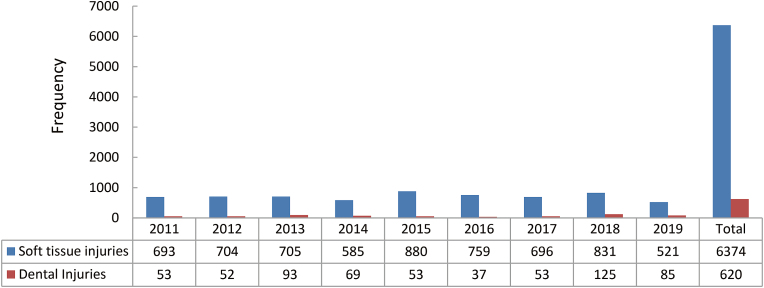
Other maxillofacial injuries.

A total of 6374 soft injuries and 620 dental injuries were the other injuries noted in the maxillofacial regions in the current study. Details are depicted in Fig. 8.

### 3.5 Facial injury distribution over time

**Fig. 9:**
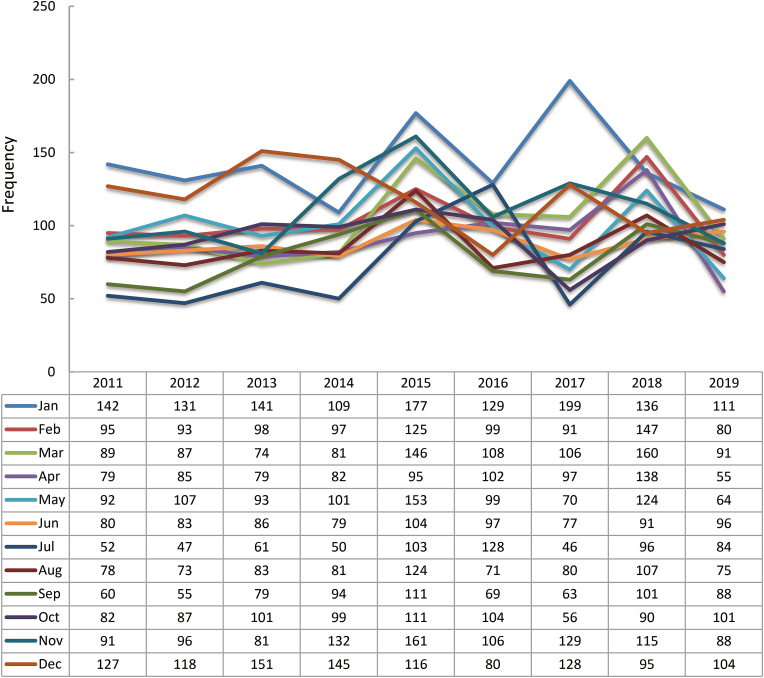
Monthly distribution of facial injuries *vs.* yearly distribution.

The 9-year retrospective study analysis reported a progressive lessening in the reported cases among the 7 of the 12 months i.e., January, February, April, May, August, November, and December. A reportable ascent in the cases was noted in March, June, July, September, and October. Surprisingly, the collective data reported a contradictory outcome. The highest cases were seen in January (*n* = 1275) and December (*n* = 1064); whereas the least cases of injuries were reported in July (*n* = 667) and September (*n* = 720). The details of monthly and seasonal distribution are demonstrated in Fig. 9.

**Fig. 10:**
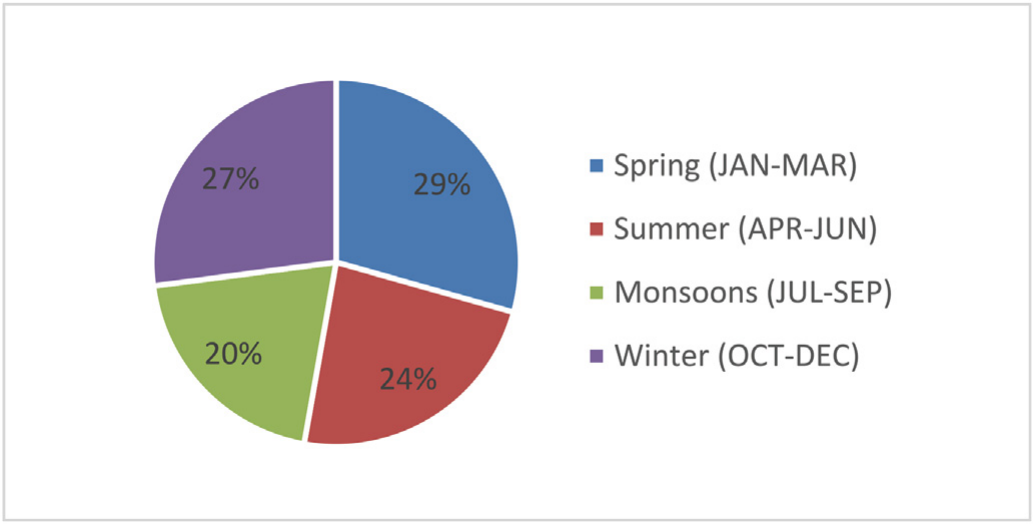
Seasonal variations.

The most significant percentage of maxillofacial injuries were noted in spring (*n* = 3142, 29.4%), followed by winter (*n* = 2894, 27.0%). The season with the least number of cases was the Monsoons (*n* = 2159, 20.2%). The details of seasonal distribution are shown in Fig. 10.

## 4 Discussion

The global variation in the distribution of maxillofacial injuries is noted in terms of demographic factors (age and gender), topography, community, ethnicity, and habitat. Our study reported the pattern of maxillofacial injuries in the Goan population for 9 years from 2011 to 2019. The study was planned to be conducted over a decade. However, due to the outbreak of the Coronavirus pandemic, data collection was restricted to 9 years to avoid bias. This study was carried out mainly to understand the changing trend of maxillofacial injuries over the years.

The maximum maxillofacial injuries were reported in the age group between 19 and 35 years, with a marked elevation in the incidence rate up to 57.6%. This was in parallel with previous published literature.^1^^,^^5^, ^6^, ^7^ This is interconnected with the dynamic nature of this group to engage in adventurous, dangerous, and extraneous activities. Despite a prominent rise in 3 of the instances, i.e., years 2013, 2015, and 2018, there was a progressive decrease noted over the years in this age group, with the least cases noted in 2019. These may be attributed to introduction and implementation of the new stringent traffic rules and regulations for the benefit of the general public. Motor Vehicles Act was implemented to improve road safety, impose huge fines and penalties on the offenders for various traffic related offences (over speeding, dangerous driving, drunken driving, driving despite disqualification, driving without insurance, driving without helmets, and blocking emergency vehicles) as well as compensation for death and grievous injury in RTA. The guardian would be deemed guilty in case of road offence by juveniles, charged penalty and the registration of the motor vehicle would be canceled. The least affected group belonged to the age group of 1 – 7 years (2.6%). This is associated with the elasticity of the paediatric bones, the smaller face to head size ratio and minimal exposure to major trauma.^8^^,^^9^

A consistent male predominance was noted throughout the study period with 80.7% of affected males, which was analogous to the study by Kumar et al.^5^ from the same centre and existing literature.^1^^,^^6^^,^^10^^,^^11^ A possible explanation is the higher involvement of males in casualties resulting from RTAs, falls, assaults, occupational accidents, and sports. However, the male to female ratio (M: F = 4:1) revealed a considerable drop, owing to escalation in the affected female population. This was in divergence with the previous study by Kumar et al.^5^ (M: F = 6:1) but comparable to the findings of Rezaei et al.^10^ (M: F = 3.5:1) and Bali et al.^7^ (M: F = 4.2:1). An increase in independence among women leading to a larger ratio of them stepping out and commuting to their workplaces, and personally driving their own vehicles may be a contributory factor along with a mild ascent in reported cases of domestic abuse.

Globally, the principal causes of maxillofacial injuries include RTAs, falls, and assaults, which is also witnessed in the present study. This is consistent with most of the reported literature.^11^^,^^12^ A 4.5% increase in RTAs incidence from 76.0%^5^ to 80.5% (in the present study) was observed. Unawareness or lack of road safety measures, bad or poor maintenance of roads, violation of traffic rules, clunker vehicles, and substance abuse along with increased traffic on roads are some of the prime reasons for an increase in RTAs.^1^^,^^5^^,^^6^^,^^13^ RTAs, the primary cause of trauma in male population, accounted for a marked increase in the affected males in the years from 2011 to 2015, followed by a progressive decrease from the year 2015 – 2019. However, there was a noticeable increment noted in the 9-year recorded data in the overall male population. A reverse pattern was seen in females. A steady rise was recorded in the years from 2011 to 2013, 2014 – 2015, and 2016 – 2018 and a visible decline was noted in the years from 2013 to 2014 and from 2018 to 2019. The number of females affected by RTAs showed a considerable decrease from 2011 to 2019. The number of males injured by RTAs continued to be higher than females (6989:1626) over the years. The possible explanation for this may be the rasher and more daring road behaviour of males as compared to females who are generally more subdued in this regard.^14^

Falls contributed to the second most common cause of facial injuries (*n* = 1027, 9.6%). The number of males affected by falls (*n* = 827) was much greater than that of females (*n* = 200). Both genders revealed an overall reduction in the incidence from 2011 to 2019 with mixed patterns of distribution in between. Falls being a common occurrence in India are accredited to uncomfortable climate conditions and unsafe and substandard labour conditions that often lead to syncope and fatigue. The third contributory factor for facial injury was assault (*n* = 860, 8.0%). A decreased incidence of this factor was noted compared to other countries, where assault is considered as a leading cause.^15^, ^16^, ^17^ Our study reported assaults as the secondary etiological factor in female population, accounting for 10.6% of the total affected females. But the overall pattern was quite contradictory. The number of reported cases of assaults in 2019 was significantly lesser than in the preceding years, owing to probably the enhanced amendments laid down for the protection of the victim, and stricter actions against the offender.

Urbanization with a rapid increase in construction of better roads and bridges is currently being witnessed in developing countries like India. As such, road safety has taken a hit. Lack of awareness and understanding among the majority of uneducated people in developing countries also contribute to an increase in RTAs. In contrast, assaults and sports injuries are seen at a higher frequency in developed countries where domestic and other public altercations are a more common issue.^18^, ^19^, ^20^

The nasal bones are the most susceptible midface fractures following trauma. This is due to prominent nature of the nose, decreased soft tissue to bone ratio, and fragility of the bone, making it an easy target. Our study, however, reported very few cases, which occurred concomitantly with panfacial trauma. Hence, we did not list them as a separate entity.

The unusual finding of our study was a remarkable change in the fracture distribution. Our study reported a considerably higher range of midface fractures (*n* = 2953, 52.5%), followed by the mandible (*n* = 2141, 38.1%). This was in contrast with previous publications^3^^,^^6^^,^^7^, which is supported by the high velocity nature of accidents coupled with poor protective measures. The zygoma continued to be the most fractured bone in the midface (84.5%), mainly due to its prominent anatomy. The most common fracture in the mandible was the condylar fracture (26.9%). This finding is in agreement with previous literature^21^^,^^22^, which could be explained again, due to the anatomy of the region, as well as a suggested protective mechanism to prevent further injury to the base of the skull.

The maximum number of cases were seen in the spring season (January to March), which is different than that mentioned in the literature.^10^^,^^18^ The second was the winter season (October to December), coincident with the tourist season in Goa.

Overall, our study has described a considerable rise in the trauma cases reported till 2015, due to the more casual and relaxed attitude of public towards fulfilment of law and order. However, from 2015, there has been a clear decline in the reported cases. The enactment of stricter rules and regulations along with use of safety gears may have led to this decrease. The fewest number of affected people was noted in 2019.

The strength of this study is the large sample size and long time frame of observation, conducted in the sole tertiary care centre of the state. There were some limitations of this study. Firstly, the retrospective nature of the study increases the likelihood of bias occurring secondary to imprecise documents or partial examination due to the manual extraction of data. Secondly, the study was uni-centric. Thirdly, neither the details on alcohol consumption nor the use of helmets were included. Finally, we also excluded the treatment methods as it is beyond the scope of this article.

Prevention is the single most effective method of preventing trauma. Safety measures such as use of helmets and seat belts, stringent laws on airbags and other protective car features, lowering of speed limits and adequate road signs and diversions in the midst of construction could aid in reducing the frequency of RTAs. In addition, road safety education should be instilled among adolescents and violation of road laws should be strictly penalised. The trauma load due to other etiology should also be combatted by better care of geriatric patients, more organized workplaces, and harsh laws for interpersonal violence.

In conclusion, this retrospective epidemiological study conducted in a sample size of 10,703 patients with 5623 fractures, 6374 soft injuries and 620 dental injuries describes a change in the incidence of the injuries along with variation in the demographic data. A marked increase of 57.6% in the incidence was recorded in the age group of 19 – 35 years affected by the maxillofacial injuries. The striking outcome was a considerable decrease in the overall male to female ratio in the 9-year cumulative data analysis, owing to the increased incidence of the affected female population. However, the male predominance in the overall population was consistent. A 4.5% increase in RTAs incidence was observed in our study, with RTAs still retaining the highest contributory factor affecting both genders. There were fewer reported cases of assault over the decade, owing to the enhanced amendments laid down for the protection of the victim, and stricter actions against the offender, despite it being the secondary cause of trauma in female population. The most unusual finding of our analysis was a remarkable change in the fracture distribution from mandible to midface. However, the zygoma and the condyle continue to be the most fractured bone in the midface, and mandibular region respectively. The implementation of safety gears, reinforcement of stricter traffic laws along with awareness may aid in the overall reduction of maxillofacial injuries.

## Funding

Nil.

## Ethical statement

This article has been approved by the local ethical committee (No. GDCH/IEC/VII-2022(04)-PROV).

## Declaration of competing interest

Authors declare that they have no conflict of interest.

## Author contributions

Purva Vijay Sinai Khandeparker: conception of study design, acquisition, analysis and interpretation of data, and drafting and final approval of the manuscript; Trishala Bhadauria Fernandes: acquisition of data, and drafting, critical revision and final approval of the manuscript; Vikas Dhupar: conception of study design, analysis and interpretation of data, and final approval of the manuscript; Francis Akkara: conception of study design, and critical revision and final approval of the manuscript; Omkar Anand Shetye: acquisition analysis and interpretation of data, and critical revision of the manuscript; Rakshit Vijay Sinai Khandeparker: analysis and interpretation of data, and critical revision of the manuscript.
